# A Rare Presentation of Intracerebellar Schwannoma: A Case Report

**DOI:** 10.1155/2024/8678186

**Published:** 2024-10-16

**Authors:** Mohamed Alhantoobi, Nadeen Alkhoori, Hassan Khayat, Euan Zhang, Almunder Algird, John Provias

**Affiliations:** ^1^Department of Neurosurgery, Hamilton General Hospital, McMaster University Medical Centre, 237 Barton St E., Hamilton, Ontario, Canada L8L 2X2; ^2^Department of Neurosurgery, Zayed Military Hospital, Abu Dhabi, UAE; ^3^Department of Radiology, Hamilton General Hospital, McMaster University Medical Centre, 237 Barton St E., Hamilton, Ontario, Canada L8L 2X2; ^4^Department of Pathology & Molecular Medicine, Hamilton General Hospital, McMaster University Medical Centre, 237 Barton St E., Hamilton, Ontario, Canada L8L 2X2

**Keywords:** intracerebellar cystic lesion, neurofibromatosis, schwannoma

## Abstract

**Background:** Intracerebellar schwannoma is an extremely rare disease entity with only 21 case reports described in the literature.

**Case Description:** A 68-year-old male presented with chronic headaches, dizziness, gait imbalance, and incoordination. Previous MRI had revealed a cystic lesion in the right cerebellum; however, patient was lost to follow-up. Updated MRI revealed dramatic enlargement of the lesion in addition to worsening clinical status. The patient underwent successful surgical resection.

**Conclusion:** Intracerebellar schwannoma can be challenging to diagnose preoperatively due to its rare occurrence; however, it should be included in the differential diagnosis of cystic lesions in the cerebellum, and most cases can be successfully treated with complete surgical resection. Pathological examination revealed a spindle cell neoplasm with other typical histopathological features of schwannoma.

## 1. Introduction

Cystic cerebellar lesions present a diagnostic challenge due to the overlap between diverse etiologies. Even though the differential diagnosis list may include intracerebellar schwannoma, it remains a very rare entity with only 21 reported cases of cerebellar schwannomas documented to date in the English literature [1]. While cystic changes are a common feature of classical schwannomas, the presence of a cystic cerebellar lesion typically prompts consideration of a broader differential diagnosis, including more common entities such as hemangioblastomas or metastatic lesions [1]. The rarity of intracerebellar schwannomas, coupled with their nonspecific radiological appearance, can lead to diagnostic uncertainty and potential misdiagnosis. Furthermore, the absence of a family history of neurofibromatosis in this patient further underscores the uniqueness of this case and the potential for sporadic occurrence of these rare tumors [1]. In this case study, we report a case of a cystic intracerebellar schwannoma in a patient without any family history of neurofibromatosis.

## 2. Case Presentation

**Figure 1:**
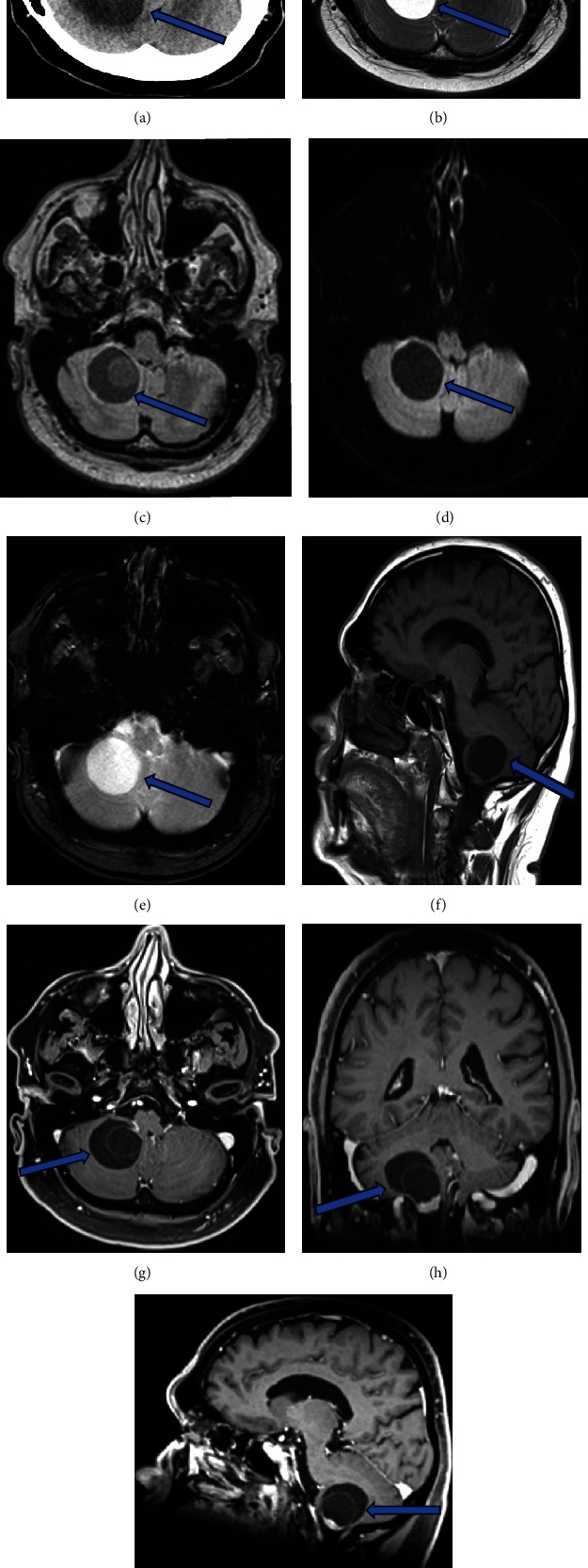
Right intracerebellar cystic lesion. (a) Axial noncontrast head CT shows a simple intra-axial cystic mass within the right cerebellum. The content is mildly heterogeneous. There is mild local mass effect effacing the fourth ventricle and causing slight tonsillar herniation with no vasogenic edema or obstructive hydrocephalus. (b) Axial T2-weighted image confirms the thin-walled cystic nature of the mass but also reveals a cyst-within-a-cyst element. (c) FLAIR demonstrates a hyperintense signal of the cyst-within-a-cyst which indicates slight complexity of the fluid content. (d) ADC MRI reveals no diffusion restriction of the fluid content which excludes epidermoid cyst. (e, f) T1 images lack susceptibility and hyperintense signal which rules out hemorrhage. (g, h, i) 3D T1-post gadolinium images reveal a small nodular component at the inferior aspect of the mass, associated with the cyst-within-a-cyst.

A 68-year-old male with a known history of prostatic hyperplasia presented with chronic headaches, dizziness, gait imbalance, and incoordination since October 2022. Three years ago, a brain MRI had revealed a cystic lesion in the right cerebellum, but due to the COVID pandemic and stable neurological condition, no further follow-up was conducted. However, a recent head CT followed by brain MRI demonstrated an increase in the size of the lesion, prompting referral to our institution (Figure 1).

Upon neurological examination, positive Romberg sign, abnormal coordination, dysmetria, and sensory deficits were observed. The patient exhibited left V1 V2 hypoesthesia, mild pronator drift on the right side, and left dysmetria. The gait examination revealed abnormal tandem walk.

MRI findings revealed a lesion in the right posterior fossa with a solid and cystic component, making it challenging to determine its exact nature. The differential diagnosis included ependymoma, hemangioblastoma, and other lesions with CSF trapping. Surgical intervention was deemed necessary to establish a definitive diagnosis and provide therapeutic management.

During the suboccipital craniotomy, intraoperative inspection revealed moderate swelling of the tumor surface. The drainage of the cystic component resulted in drainage of brownish fluid, indicating a history of intracystic bleeding.

**Figure 2:**
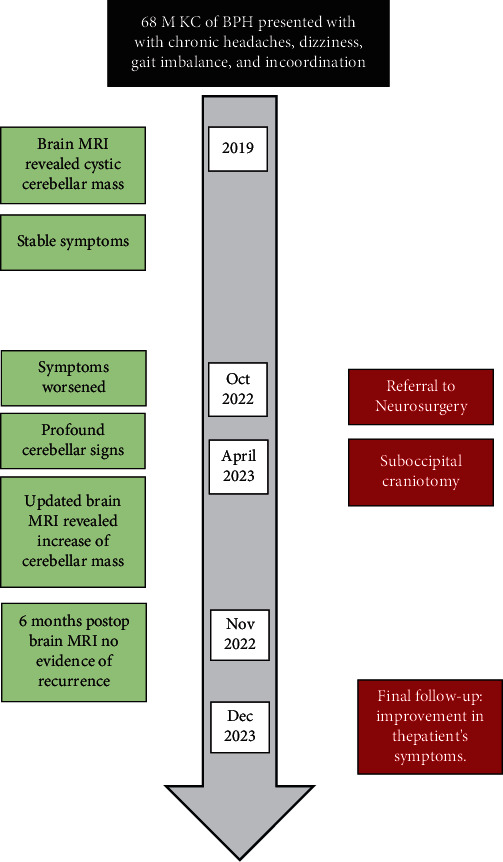
Case timeline.

Postoperative follow-up demonstrated an improvement in the patient's symptoms, including headache relief and enhanced gait stability. The patient made a remarkable recovery with no evidence of neurological deficits following the surgical resection of the intracerebellar schwannoma. Postoperative brain MRI with gadolinium contrast enhancement performed 6 months after the surgery showed stable postoperative changes and no evidence of tumor recurrence.  Figure 2 summarizes the timeline of this case.

**Figure 3:**
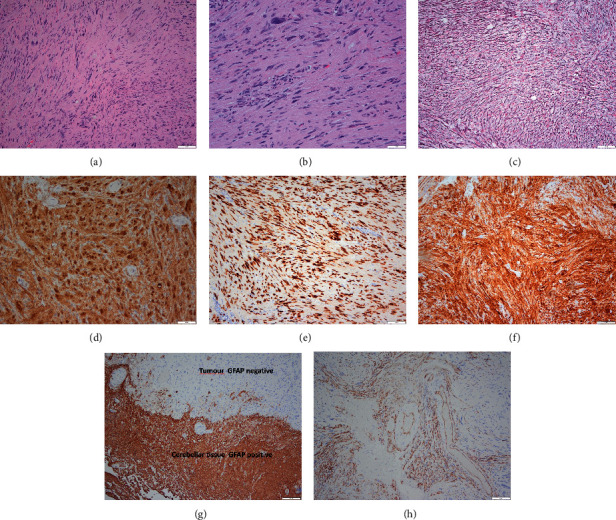
Intracerebellar region tumor pathology. Microphotographs of the biopsied tissue shows spindled cell neoplasm with fascicular architecture and vague nuclear alignment (a, b) (H/E) focal degenerative atypia (b) (H/E). Reticulin showing extensive deposition around tumor cells characteristic of schwannomas. (c) Reticulin showing extensive deposition around tumor cells characteristic of schwannomas. Immunohistochemistry (d, e, f) showing the tumor to be strongly positive for S100 (d), SOX 10 transcription factor (nuclear (e)), and P16 (f). Immunohistochemistry (g, h) showing the tumor to have a well-defined border with adjacent cerebellar tissue (g) (GFAP) and focally positive for CD34 (h). Negative IHC included EMA, melanoma cocktail, inhibin, AE1/AE, keratin, and neurofilament.

The specimen showed a spindle cell neoplasm with typical features of a schwannoma (Figure 3). Elongate spindled tumor cells with ill-defined borders, fascicular architecture, and some nuclear alignment. The tumor was predominantly Antoni type A tissue. There was a well-demarcated border with adjacent cerebellar tissue. There was focal “ancient” type nuclear degenerative change, but no atypia or mitotic activity. Proliferation assessed by Ki 67 IHC was low averaging less than 2%. The full IHC and special stain profile was typical for schwannomas with strong S100 and SOX 10 positivity (SOX 10 nuclear) patchy CD34 positive with extensive pericellular reticulin deposition. The tumor was immunonegative for EMA, GFAP, IDH 1, inhibin, neurofilament, AE1/AE3, and melanoma cocktail.

## 3. Discussion

Intraparenchymal schwannomas are rare tumors that have been predominantly reported in the spinal cord rather than within the brain [2]. The first intracranial parenchymal schwannoma was described in 1965 [3]. This discussion aims to expand on intraparenchymal schwannomas and its pathogenesis, with a focus on cerebellar schwannomas. Literature review reveals that brain parenchymal schwannomas are primarily located in the upper tentorium, with only 21 cases of cerebellar schwannoma reported to date in the English literature [2].  Table 1 illustrates an updated summary of all reported cases of intracerebellar schwannomas. The histogenesis of intracerebral schwannomas remains controversial, and multiple theories have been proposed to explain their occurrence. One theory suggests that schwannomas arise from the proliferation of Schwann cells within perivascular nerve plexi ([20, 21]. These minute nerves may be present in the tela choroidea, the embryological precursor of the choroid plexus, which could explain the proximity of many tumors to the ventricular system. Furthermore, Schwann cells have been identified in subarachnoid space, and the peripheral areas of old cerebral infarcts and multiple sclerosis plaques. The occurrence of Schwann cells in these conditions may be attributed to proliferating multipotent mesenchymal cells, rather than mature Schwann cells accompanying perivascular plexi [22]. Another hypothesis suggests that intracerebral schwannomas may arise from neural crest elements displaced during embryogenesis [23]. The distribution and dynamics of stem cells in the central nervous system have not been fully elucidated, which further complicates the understanding of intracerebellar schwannoma pathogenesis. To gain a comprehensive understanding, detailed morphological analysis of intact tumors and surrounding tissues is crucial.

Cystic tumors can develop as a result of different factors including microhemorrhage, necrosis, or degeneration. These factors can result in the formation of a small cavity, followed by the formation of a continuous capsule [8, 23]. Most of these tumors are benign, and their clinical symptoms are typically associated with the specific location within the body. However, it is important to note that the clinical symptoms and imaging characteristics of these tumors are not distinctive, necessitating the use of both histological examination and immunohistochemical studies to confirm a diagnosis of brain parenchymal schwannoma.

Immunohistochemistry can reveal specific markers for this type of tumor, such as S100 protein (+), vimentin (+), and glial fibrillary acidic protein (GFAP) (−), among others [16, 24]. It is worth mentioning that brain parenchymal schwannomas are located within the brain tissue itself and do not exhibit any apparent association with the surrounding dura or cranial nerves.

This case report provides an opportunity to compare and contrast with previously documented cases, shedding light on the variability in clinical presentations, radiological features, and treatment outcomes associated with these uncommon tumors [1]. Unlike many previously reported cases that presented with symptoms of increased intracranial pressure or focal neurological deficits, this patient's initial presentation with dizziness and nausea was relatively nonspecific [1, 15, 17–19]. This highlights the diagnostic challenge posed by intracerebellar schwannomas, as their symptoms can mimic more common pathologies, such as vestibular disorders or cerebellar infarcts [1, 15, 17–19].

Interestingly, the absence of a family history of neurofibromatosis type 2 (NF2) in this case contrasts with several previously reported cases, where intracerebellar schwannomas were associated with this genetic condition [1, 24, 25]. This underscores the potential for sporadic occurrence of these tumors, further complicating the diagnostic process [25].

The radiological appearance of the cystic cerebellar lesion in this case was initially suggestive of a broader differential diagnosis, including hemangioblastoma or metastasis. This overlap in imaging features with more common pathologies is consistent with the experience reported in previous cases, highlighting the limitations of relying solely on radiological findings for a definitive diagnosis [19, 25]. Consistent with the standard management approach for intracerebellar schwannomas, the authors pursued complete surgical resection, which is generally associated with favorable outcomes [18, 19]. The patient's uneventful postoperative recovery and the absence of tumor recurrence on follow-up imaging align with the positive outcomes reported in several previous cases where complete resection was achieved [1, 18, 19]. However, it is important to note that some reported cases have experienced postoperative recurrence or residual tumor growth, necessitating additional interventions or close follow-up ([1, 24, 25] [10]; [9]). This variability in outcomes underscores the need for long-term surveillance and the potential role of adjuvant therapies, such as radiation therapy, in select cases [24].

In summary, this case report contributes to the growing body of literature on intracerebellar schwannomas by highlighting the diagnostic challenges, variable clinical presentations, and the importance of complete surgical resection for optimal outcomes. By comparing and contrasting with previously reported cases, the authors provide valuable insights into the management of these rare tumors and emphasize the need for continued research to improve diagnostic accuracy and treatment strategies.

## 4. Conclusion

Intraparenchymal schwannomas are rare tumors that are more commonly found in the spinal cord than in the brain. The pathogenesis of these tumors remains controversial, with theories involving Schwann cell proliferation, neural crest displacement, and metaplastic changes in soft membrane cells [1, 2, 22]. Further research and detailed morphological analysis are necessary to fully understand the pathogenesis of intracerebellar schwannomas [1, 16]. Nonetheless, most cases can be successfully treated with complete surgical removal, and the prognosis is generally favorable, without the need for additional radiotherapy or chemotherapy [1].

## Figures and Tables

**Table 1:** Updated summary of previously reported cases of cerebellar schwannoma [1].

**Authors**	**Age (years)**	**Sex**	**Tumor location**
Kuhn, Neely, and Pollay [4]	42	F	Cerebellar vermis
Sarkar, Mehta, and Roy [5]	24	M	Cerebellar hemisphere
Schwartz and Sotrel [6]	48	M	Cerebellar hemisphere
Tran-Dinh et al. [7]	64	F	Cerebellar vermis
Chitre et al. [8]	35	F	Cerebellar vermis
Casadei et al. [2]	52	F	Cerebellar hemisphere
Casadei et al. [2]	55	M	Cerebellar hemisphere
Casadei et al. [2]	79	F	Cerebellar vermis
Sharma et al. [9]	73	F	Cerebellar vermis
Sharma et al. [10]	45	M	Cerebellar vermis
Sharma et al. [10]	24	M	Cerebellum
Ranjan Chacko, and Chandi [11]	65	F	Cerebellar hemisphere
Tanabe et al. [12]	68	F	Cerebellar hemisphere
Tsuiki et al. [13]	64	F	Cerebellar hemisphere
Bhatjiwale and Gupta [14]	15	M	Cerebellar vermis
Jabbour et al. [15]	9	F	Cerebellar hemisphere
Maiuri et al. [16]	29	F	Cerebellar vermis
Chung, Cherian, and Chandran [17]	49	F	Cerebellar hemisphere
Umredkar, Gupta, and Radotra [18]	35	F	Cerebellar vermis
Xuejian et al. [19]	52	F	Cerebellar hemisphere
Takeuchi et al. [1]	61	M	Cerebellar hemisphere
Present case	68	M	Cerebellar hemisphere

## Data Availability

Some data are available upon request due to ethical considerations. Interested researchers can contact the corresponding author at Mohamed.alhantoobi@medportal.ca to request access to the data. Data will be shared in accordance with institutional guidelines and after obtaining necessary permissions.

## References

[1] Takeuchi Y., Arakawa Y., Yokoo H. (2022). Intra-cerebellar schwannoma with various degenerative changes: a case report and a systematic review. *BMC Neurology*.

[2] Casadei G. P., Komori T., Scheithauer B. W., Miller G. M., Parisi J. E., Kelly P. J. (1993). Intracranial parenchymal schwannoma. *Journal of Neurosurgery*.

[3] David M., Guyot J. F., Ballivet J., Sachs M. (1965). Tumeur Schwannoide Du Ventricule Latrral. *Neuroehirurgie*.

[4] Kuhn J. R., Neely J. G., Pollay M. (1985). Cystic cerebellar schwannoma. *Otolaryngology–Head and Neck Surgery*.

[5] Sarkar C., Mehta V. S., Roy S. (1987). Intracerebellar schwannoma. *Journal of Neurosurgery*.

[6] Schwartz A. M., Sotrel A. (1988). Intracerebral and intracerebellar neurilemoma. *Southern Medical Journal*.

[7] Tran-Dinh H. D., Soo Y. S., O’Neil P., Chaseling R. (1991). Cystic cerebellar schwannoma: case report. *Neurosurgery*.

[8] Chitre M. B., Rajshekhar V., Chandi S. M., Chandy M. J. (1992). Cystic cerebellar schwannoma. *British Journal of Neurosurgery*.

[9] Sharma R. R., Gurusinghe N. T., Lynch P. G., Parekh H. C., Bertolis G. (1993). Intraparenchymatous schwannoma of the cerebellum. *British Journal of Neurosurgery*.

[10] Sharma M. C., Karak A. K., Gaikwad S. B., Mahapatra A. K., Mehta V. S., Sudha K. (1996). Intracranial intraparenchymal schwannomas: a series of eight cases. *Journal of Neurology, Neurosurgery & Psychiatry*.

[11] Ranjan A., Chacko G., Chandi S. M. (1995). Intracerebellar melanotic schwannoma: a case report. *British Journal of Neurosurgery*.

[12] Tanabe M., Miyata H., Okamoto H. (1996). Brainstem schwannoma —case report—. *Neurologia Medico-Chirurgica*.

[13] Tsuiki H., Kuratsu J., Ishimaru Y. (1997). Intracranial Intraparenchymal schwannoma: report of three cases. *Acta Neurochirurgica*.

[14] Bhatjiwale M., Gupta S. (1999). Midline cerebellar cystic schwannoma: a case report. *Neurology India*.

[15] Jabbour P., Rizk T., Lahoud G. A. (2002). Schwannoma of the tentorium cerebelli in a child. *Pediatric Neurosurgery*.

[16] Maiuri F., Colella G., D’Acunzi G., Del Basso De Caro M. (2004). Malignant intracerebellar schwannoma. *Journal of Neuro-Oncology*.

[17] Chung K. H. C., Cherian M., Nadana Chandran K. (2007). Schwannoma with tentorial attachment in the cerebellopontine angle mimicking a meningioma. *Journal of Clinical Neuroscience*.

[18] Umredkar A., Gupta S. K., Radotra B. (2011). Posterior fossa vermian cystic schwannoma mimicking as pilocytic astrocytoma: a case report and literature review. *Neurology India*.

[19] Xuejian W., Xiaobiao Z., Hu F. A. N., Yu Y. O. N. G., Gu Y. E., Xie T. A. O. (2013). Intracerebellar hemispheres schwannoma: case report. *Turkish Neurosurgery*.

[20] Ghatak N. R., Norwood C. W., Davis C. H. (1975). Intracerebral schwannoma. *Surgical Neurology*.

[21] Sung J. H., Mastri A. R., Chen K. T. K. (1981). Aberrant peripheral nerves and neuromas in normal and injured spinal cords. *Journal of Neuropathology and Experimental Neurology*.

[22] Feigin I., Ogata J. (1971). Schwann cells and peripheral myelin within human central nervous tissues. *Journal of Neuropathology and Experimental Neurology*.

[23] Gambarelli D., Hassoun J., Choux M., Toga M. (1982). Complex cerebral tumor with evidence of neuronal, glial and Schwann cell differentiation: a histologic, Immunocytochemical and ultrastructural study. *Cancer*.

[24] Gao Y., Qin Z., Li D. (2018). Intracerebral schwannoma: a case report and literature review. *Oncology Letters*.

[25] Coy S., Rashid R., Stemmer-Rachamimov A., Santagata S. (2020). An update on the CNS manifestations of neurofibromatosis type 2. *Acta Neuropathologica*.

